# Implementation and Evaluation of Virtual Anticoagulation Clinic Care to Provide Incessant Care During COVID-19 Times in an Indian Tertiary Care Teaching Hospital

**DOI:** 10.3389/fcvm.2021.648265

**Published:** 2021-03-29

**Authors:** Sunil Kumar Shambu, Shyam Prasad Shetty B, Oliver Joel Gona, Nagaraj Desai, Madhu B, Ramesh Madhan, Revanth V

**Affiliations:** ^1^Department of Cardiology, Jagadguru Sri Shivarathreeshwara Medical College and Hospital, Jagadguru Sri Shivarathreeshwara Academy of Higher Education and Research (JSS AHER), Mysore, India; ^2^Department of Cardiothoracic and Vascular Surgery, Jagadguru Sri Shivarathreeshwara Medical College and Hospital, Jagadguru Sri Shivarathreeshwara Academy of Higher Education and Research (JSS AHER), Mysore, India; ^3^Department of Pharmacy Practice, Jagadguru Sri Shivarathreeshwara College of Pharmacy (JSS CPM), Jagadguru Sri Shivarathreeshwara Academy of Higher Education and Research (JSS AHER), Mysore, India; ^4^Department of Community Medicine, Jagadguru Sri Shivarathreeshwara Medical College and Hospital, Jagadguru Sri Shivarathreeshwara Academy of Higher Education and Research (JSS AHER), Mysore, India

**Keywords:** anticoagulation clinic, vitamin K antagonist, time in therapeutic range, percentage of international normalized ratio in range, telehealth

## Abstract

**Background:** COVID-19 caused by severe acute respiratory syndrome coronavirus 2 (SARS-CoV-II) has become a global pandemic disrupting public health services. Telemedicine has emerged as an important tool to deliver care during these situations. Patients receiving Vitamin K antagonists (VKA) require structured monitoring which has posed a challenge during this pandemic. We aimed to evaluate the impact of Virtual anticoagulation clinic (VAC), a Telehealth model on the quality of anticoagulation, adverse events, and patient satisfaction vis-a-vis standard Anticoagulation clinic (ACC) care.

**Materials and methods:** A bidirectional cohort study was conducted in the Department of Cardiology, JSS Hospital, Mysore. Two hundred and twenty-eight patients in the VAC and 274 patients in the ACC fulfilling inclusion criteria were the subjects of the study. Telehealth tools like WhatsApp and telephone were used. Time in therapeutic range (TTR), Percentage of International normalized ratio in range (PINRR), and adverse events were analyzed and compared between the VAC group and the ACC group, between pre-COVID and COVID ACC groups, and between the VAC group and the same pre-COVID cohort. Patient satisfaction was assessed by a questionnaire at the end of 8 months. Descriptive statistics were used for the patient characteristics and inferential statistics for the comparisons between pre-VAC and VAC care.

**Results:** The mean TTR was 75.4 ± 8.9% and 71.2 ± 13.4% in the VAC group and ACC group, respectively (*p* < 0.001). The mean PINRR was 66.7 ± 9.4% and 62.4 ± 10.9% in the VAC group and ACC group respectively, (*p* < 0.001). There was no significant difference in TTR between the VAC group and the same pre-COVID cohort. The TTR differential between the pre-COVID and COVID ACC groups was significant. In either group, no major adverse events were seen. The most common tools used for data exchange were WhatsApp (83%) and SMS (17%). Seventy-four percent of patients were extremely satisfied with the overall VAC care.

**Conclusions:** Virtual anticoagulation clinic, a telehealth model can be used as an alternative option to deliver uninterrupted anticoagulation care during pandemic times.

## Introduction

COVID-19 caused by severe acute respiratory syndrome coronavirus 2 (SARS-CoV-II) has become a global pandemic disrupting public health services (1). In these time frames, effective clinical care for patients with various chronic cardiovascular and other disorders has gained considerable attention from various stakeholders (2). In this predicament, Telehealth a virtual platform for the care provider and seeker has great potential in providing cardiovascular care which is evidently quite ideal (3). Its utility for patients on oral anticoagulants is one domain that needs to be addressed. Of the anticoagulants, vitamin K antagonists (VKAs) have a narrow therapeutic index with variable dose-response and diet/drug interactions (4). Patients taking VKAs require International normalized ratio (INR) monitoring and dose titration to achieve therapeutic INR for optimal outcomes (5). Patients taking VKAs may have multiple comorbidities like advanced age, hypertension, diabetes mellitus, and others. Studies have shown that patients with these risk factors are susceptible to severe COVID-19 infection necessitating a strategy to mitigate exposure of such patients (6, 7).

Telehealth services help to provide patients with the necessary care while minimizing the risk of transmitting SARS-CoV-II to healthcare workers and patients (8). The notion of telemedicine was incorporated in the Anticoagulation clinic to provide uninterrupted virtual care to patients taking VKAs. This study was conducted to evaluate the impact of Virtual anticoagulation clinic care (VAC) on the quality of anticoagulation, adverse events, and patient satisfaction vis-a-vis standard ACC care.

## Materials and Methods

### Study Design and Participants

**Figure 1:**
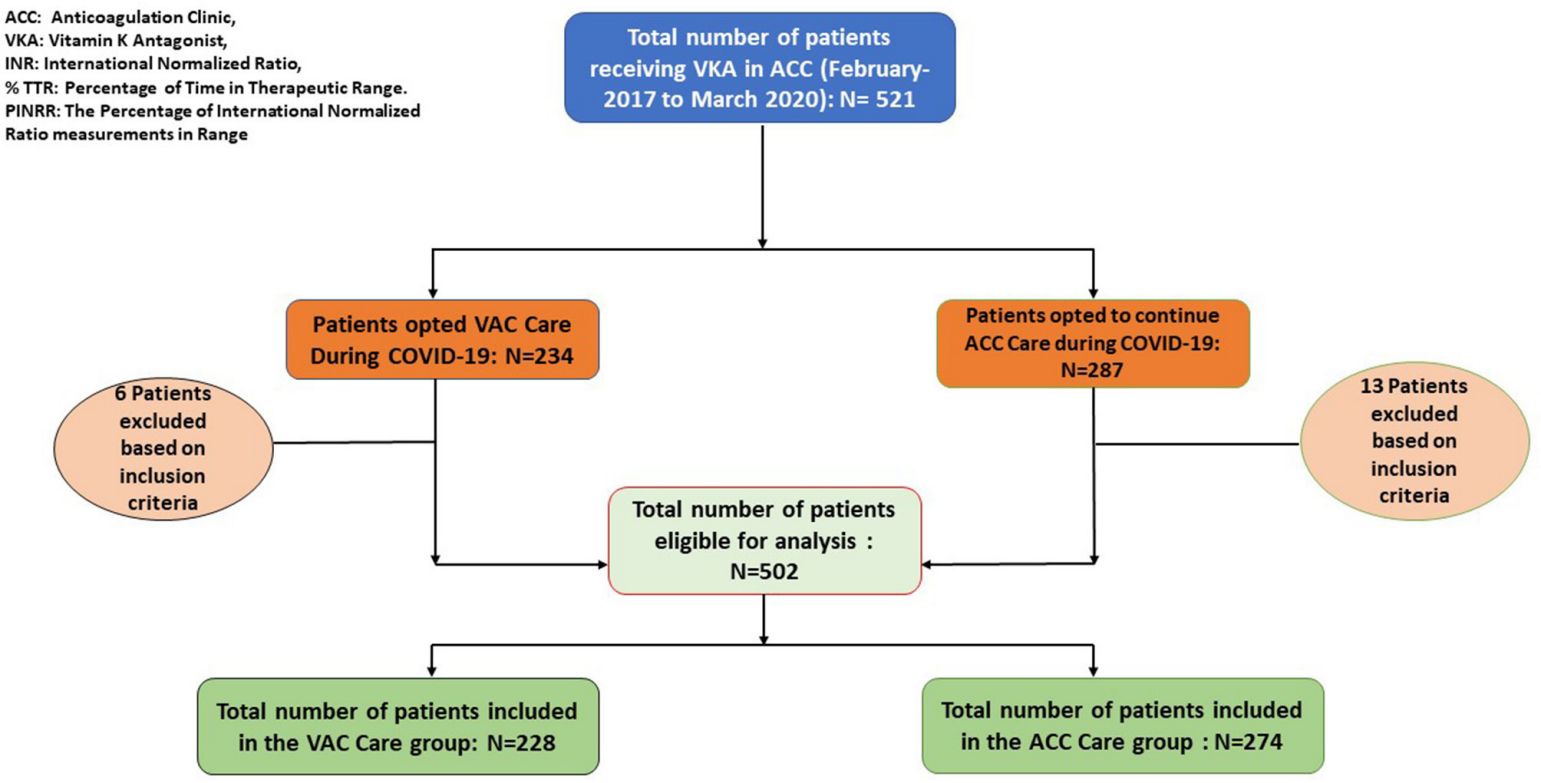
Participant enrolment process. ACC, Anticoagulation clinic; VKA, Vitamin K Antagonist; INR, International Normalized Ratio; % TTR, Percentage of Time in Therapeutic Range; PINRR, The percentage of International Normalized Ratio measurements in Range. * EXCLUSION CRITERIA: Patients having less than 3 months of ACC exposure pre COVID-19 phase and less than 3 INR values were excluded from the study.

A bidirectional observational cohort study was conducted on patients enrolled in the VAC and ACC at the Department of Cardiology, JSS Hospital, Mysore from March to November 2020. Institutional ethical committee approval was taken. A total of 521 patients were registered in ACC till March 2020. Among these, 234 patients opted for VAC care and 287 patients opted for ACC care. For calculation of TTR, patients who had more than 3 months of ACC care before March 2020 with at least 3 INR values in both groups were included in the study. Newly enrolled patients in the ACC and those patients who had less than 3 months of ACC care before March 2020 were excluded from the study. A total of 228 patients in the VAC care group and 274 patients in the standard ACC care group were eligible for analysis. The patient enrolment process is depicted in Figure 1.

### Anticoagulation Quality Assessment Tools

The anticoagulation related quality measures like Percentage Time in Therapeutic Range (%TTR) (9), Percentage of INR within Range (PINRR) (10), extreme INRs, and adverse events were analyzed. Patient satisfaction toward VAC care was assessed by administering five items self-developed questionnaire with scores 0 to 4 from extremely satisfied to not at all satisfied at the end of 8 months.

### Anticoagulation Clinic (ACC)

JSS Hospital, Mysore has an established ACC since February 2017 comprising a multidisciplinary team comprising a Senior cardiologist, Junior cardiologist, Clinical Pharmacist, Clinical Pharmacy interns, and trained nursing staff. Key issues such as patient education (VKA risks/benefits, potential diet/drug interactions), ordering relevant laboratory tests (once a month INR testing), titrating the dose of VKAs to meet the INR target, facilitating procedures requiring interruption of VKAs, and adverse effects associated with VKAs were addressed.

### Virtual Anticoagulation Clinic (VAC)

VAC was initiated in March 2020 to provide sustained care to patients taking VKAs registered in ACC during the COVID-19 pandemic. Telehealth tools like WhatsApp and telephone were used as per Telemedicine practice guidelines (11). WhatsApp and SMS were used for the asynchronous exchange of the data. Patients were supposed to undergo INR testing once a month and communicate the INR report and if any symptoms related to bleeding, Transient Ischemic Attack (TIA), or stroke by any of the tools quoting their ACC identification number. Based on the INR value, dose titration was done and advice regarding the next INR testing was given. Patients with INR <1.5 and INR >5.0, major bleeding, and systemic embolic events were advised for the hospital visit. TTR and PINRR were calculated by Rosendaal linear interpolation technique for each patient. Calculations were performed with the assistance of a template made available by INR Pro (12). Major bleeding was defined by the International Society on Thrombosis and Haemostasis criteria (13). Stroke/Systemic embolic events were defined as the combined endpoints of ischaemic stroke, TIA, and systemic embolic events.

### Statistical Analysis

Data was entered in MS Office Excel 2019 and analyzed by using IBM SPSS Statistics Version 25. Continuous variables were expressed as mean ± standard deviation (SD). Categorical variables were expressed as absolute numbers and percentages. Descriptive statistics were used for patient characteristics. *T*-test and chi-square tests (χ^2^) were used for comparisons between groups. All tests were two-tailed, *p* < 0.05 was considered to be statistically significant.

## Results

The mean age of the patients in the VAC group and ACC group was 55.62 ± 13.77 years and 53.72 ± 11.8 years, respectively. The majority of the patients in the VAC group were from rural areas (57%). On the contrary, only 30% of the patients were from rural areas in the ACC group. Patients characteristics are depicted in Table 1. Atrial fibrillation was the most common indication for VKA therapy in both groups. Acenocoumarol was the most common VKA prescribed. Mean TTR in VAC group and ACC group was 75.4 ± 8.9% and 71.2 ± 13.4%, respectively (*p*-value = 0.001). Mean PINRR in the VAC group and ACC group was 66.7 ± 9.4% and 62.4 ± 10.9%, respectively (*p*-value = 0.0002). Patients in the VAC group underwent more frequent INR testing when compared to those in the ACC group. Two patients had a minor lower gastrointestinal bleed in the VAC group. None of the patients had major adverse events in either group. Three patients were scheduled for an in-person visit in the VAC group. Anticoagulation related parameters in the VAC group and ACC group are depicted in Table 2. There was no significant difference in TTR between the VAC group and the same group during pre-COVID ACC care. There was a significant difference in TTR and PINRR between the pre-COVID and COVID-ACC groups (*p* < 0.0001). The number of INR tests performed per patient was less in the ACC group during the COVID pandemic. Anticoagulation related parameters between the groups are depicted in Table 3.

**Table 1:** Patient characteristics and anticoagulation related parameters.

**Variables**	**VAC (*N* = 228)**	**ACC (*N* = 274)**	***p*-value**
**Age (years)**			
<60	118 (51.6)	156 (57.07)	0.9077
>61	110 (48.4)	118 (42.93)	0.2208
**Gender**			
Men	129 (57)	167 (60.84)	0.3841
Women	99 (43)	107 (39.16)	
**Comorbidities**			
Type 2 Diabetes Mellitus	53 (23.4)	89 (32.54)	0.0239
Hypertension	73 (32)	90 (33.01)	0.8102
Congestive heart failure	18 (7.8)	36.1 (13.20)	0.0520
Vascular disease^#^	29 (12.5)	31 (11.32)	0.6842
**Educational status**			
Literate	162 (71.1)	247 (90.09)	<0.0001^††^
Illiterate	66 (28.9)	27 (9.9)	<0.0001^††^
**Location of residence**			
Urban	98 (43)	194 (70.82)	<0.0001^††^
Rural	130 (57)	80 (29.18)	<0.0001^††^
**HASBLED score**			
≥3	69 (30.4)	57 (20.75)	0.0132^††^
<3	158 (69.5)	217 (79.25)	0.0123^††^
**Vitamin K Antagonist**			
Warfarin	16 (7)	6 (2.36)	0.0121^††^
Acenocoumarol	212 (93)	268 (97.64)	0.0123^††^
Indications for VKA^*^			
Atrial fibrillation	137 (60)	192 (70.28)	0.0159^††^
Mechanical Valve replacement	8 (3.4)	44 (16.03)	<0.0001^††^
Deep vein thrombosis / Pulmonary embolism	80 (35.1)	38 (13.69)	<0.0001^††^
Cortical venous thrombosis	3 (1.5)	0	^−^

# Vascular disease: Coronary artery disease, Peripheral arterial disease;

* *VKA: Vitamin K antagonist*.

†† *statistically significant p-value has been obtained by performing chi-squared test*.

**Table 2:** Anticoagulation related Quality Parameters.

**Variables**	**VAC (*N* = 228)**	**ACC (*N* = 274)**	***p*-value**
Number of INR^**†**^ draws (1,544)	1,324	1,019	-
Average number of INR^**†**^ draws/ Patient	5.8	3.72	-
Mean TTR%^**††**^	75.4 ± 8.91	71.2 ± 13.4	0.0018^†^
Mean PINRR %^**^	66.7 ± 9.4 %	62.4 ± 10.9%	0.0002^†^
Tests Over Range	129 (9.7%)	113 (11.11%)	0.2660
Tests Below range	151 (11.7%)	142 (13.9%)	0.1124
**Extreme INRs**
INR >5.0	14 (1.06)	15 (1.51)	0.3323
INR <1.5	30 (2.26)	75 (7.32)	<0.0001
**Adverse events**
Major	0 (0%)	0	-
Minor bleeding	2 (0.8%)	0	

† INR: International Normalized Ratio;

** PINRR: Percentage of International Normalized Ratio in the Therapeutic Range;

†† *TTR: Time in Therapeutic Range*.

†† *statistically significant p-value has been obtained by performing chi-squared test*.

† *statistically significant p-value has been obtained by performing t-test*.

**Table 3:** Assessment of Anticoagulation parameters among Pre-VAC (pre COVID) and VAC care.

**Anticoagulation parameters**	**VAC care (*n* = 228)**	**Pre-COVID care (*n* = 228)**	***p*-value**	**ACC COVID-19 care (*n* = 274)**	**ACC pre-COVID care (*n* = 274)**	***p*-value**
Number of INR^**†**^ draws	1,324	1,467		1,019	1,551	
Average number of INR^**†**^ draws/ Patient	5.8	6.43	-	3.72	5.66	-
Mean TTR%^*^	75.4 ± 8.91	77.58 ± 8.85	0.0506^#^	71.2 ± 13.4	79.12 ± 9.3	<0.0001^#^
Mean PINRR %^**^	66.7 ± 9.4 %	69.68 ± 11.50	0.0241^#^	62.4 ± 10.9%	67.8 ± 10.4	<0.0001^#^
Tests Over Range	129 (9.7)	118 (8.1)	0.1375	113 (11.11)	96 (6.2)	<0.0001^††^
Tests Below range	151 (11.7)	106 (7.26)	0.0001^††^	142 (13.9)	129 (8.3)	<0.0001^††^
**Extreme INRs**						
INR >5.0	14 (1.06)	0	-	15 (1.51)	10 (0.66)	0.0339^††^
INR <1.5	30 (2.26)	11 (0.75)	0.0009^††^	75 (7.32)	8 (0.5)	<0.0001^††^
**Adverse events**						
Major	0 (0%)	0	-	0	0	-
Minor bleeding	2 (0.8%)	0		0	0	

† INR: International Normalized Ratio;

** PINRR: Percentage of International Normalized Ratio in the Therapeutic Range

* *TTR: Time in Therapeutic Range*.

†† *statistically significant p-value has been obtained by performing chi-squared test*.

# *statistically significant p-value has been obtained by performing t-test*.

**Figure 2:**
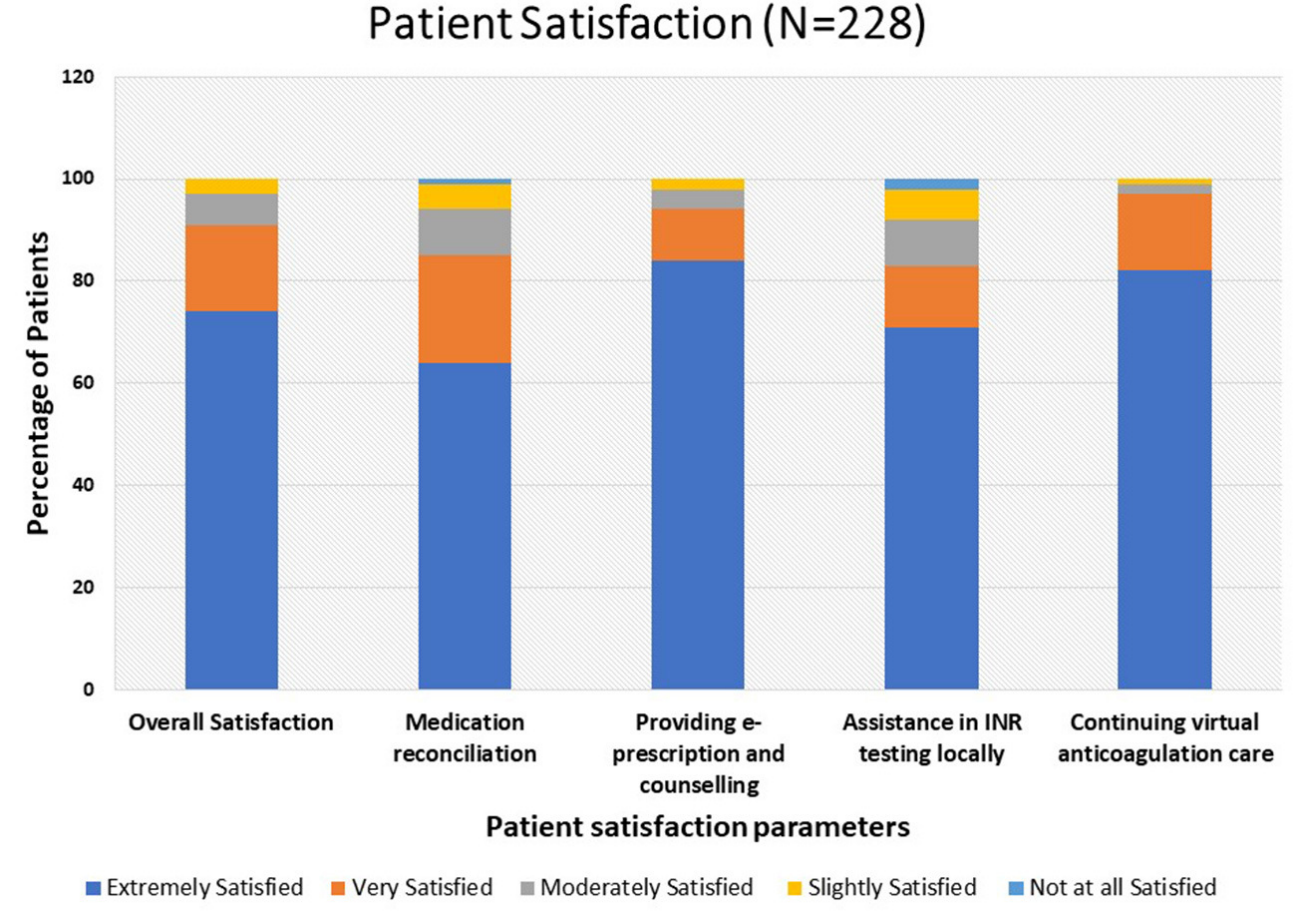
Patient satisfaction toward VAC care during COVID-19.

WhatsApp 189 (83%), followed by SMS 39 (17%) were the most common tools used for the exchange of data. One hundred and sixty-nine (74%) of patients were extremely satisfied with overall VAC care and 187 (82%) of patients were extremely satisfied to continue virtual care as assessed by a 5-item questionnaire. The patient satisfaction score and questionnaire are depicted in Figure 2 and Table 4.

**Table 4:** Patient satisfaction toward virtual anticoagulation care (VAC) during COVID-19 pandemic (*N* = 228).

**S. No**	Parameter^*^		Response^**^n (%)
1.	Overall satisfaction of patients on VAC care during COVID 19	Extremely satisfied (4)	168 (74)
		Very satisfied (3)	39 (17)
		Moderately satisfied (2)	14 (6)
		Slightly satisfied (1)	7 (3)
		Not at all satisfied (0)	(0)
2.	Medication	Extremely satisfied (4)	146 (64)
	reconciliation	Very satisfied (3)	48 (21)
		Moderately satisfied (2)	20 (9)
		Slightly satisfied (1)	12 (5)
		Not at all satisfied (0)	2 (1)
3.	Providing e-prescription	Extremely satisfied (4)	192 (84)
	and education	Very satisfied (3)	23 (10)
	reinforcement	Moderately satisfied (2)	8 (4)
	(counseling)	Slightly satisfied (1)	5 (2)
		Not at all satisfied (0)	(0)
4.	Assistance in INR	Extremely satisfied (4)	162 (71)
	monitoring locally	Very satisfied (3)	27 (12)
	despite lockdown	Moderately satisfied (2)	20 (9)
	during COVID 19	Slightly satisfied (1)	14 (6)
		Not at all satisfied (0)	5 (2)
5.	Continuing virtual	Extremely satisfied (4)	187 (82)
	anticoagulation care	Very satisfied (3)	34 (15)
		Moderately satisfied (2)	5 (2)
		Slightly satisfied (1)	2 (1)
		Not at all satisfied (0)	0

* *Feedbacks for Q1 – Q7 were obtained through a 5-point Likert scale with scoring 0 – 4, 0 = Not at all Satisfied, 1 = Slightly Satisfied, 2 = Moderately Satisfied, 3 = Very Satisfied, 4 = Extremely Satisfied*.

** *Data represented as frequency and proportion*.

## Discussion

In our study, the principal findings were (1) Patients in the VAC group had greater control of anticoagulation in the form of more time spent in the therapeutic range compared to ACC during the COVID pandemic (75.4 and 71.2%, respectively). (2) There was no significant difference in TTR between the VAC group and the same patients in the Pre-COVID ACC care 3). There was a significant difference in TTR between the pre-COVID and COVID ACC groups.

Due to the COVID pandemic, healthcare was inaccessible to the majority of the patients. Telehealth-based VAC initiated during that period could deliver uninterrupted care to the patients on chronic VKA therapy. Patients in the virtual care group could maintain their mean TTR similar to that of ACC care during the pre-COVID state. Wherein patients in the ACC care group were unable to maintain the mean TTR because of less frequent INR testing and in-person visits. Similar telehealth-based studies conducted on patients with chronic warfarin therapy have reported mean TTRs ranging from 66 to 74% (14–16).

Several meta-analyses of randomized and real-world trials have found that TTRs and PINRRs are generally equal to or below 60% (10, 17, 18). The European consensus document recommends a TTR of >70% for optimal outcomes (19). NICE guidelines recommend a TTR of > 65% for patients with AF on VKA therapy (20). In our study, achieved TTRs in both groups were above the proposed benchmark of >65–70%. One of the main reasons to achieve mean TTR > 70% in our study was because our cohort of patients were those registered in the ACC managed by a multidisciplinary team. Even randomized controlled trials and studies related to Anticoagulation clinics have documented better control of INR compared to community settings that were possible due to frequent monitoring, organized care, and improvement in adherence to VKAs (10, 17).

Other important and desirable points to note were that these patients had multiple comorbidities and could be treated with the reduced risk of exposure to COVID-19 infection during transit to the hospital, cost savings for travel, and no major adverse events. The majority of the patients were satisfied with overall virtual care and opted for virtual care even in the post-COVID state.

The tenable reasons for the patients to continue to benefit from following up in VAC are several. Patients were educated during their initial visits to the regular anticoagulation clinic about the importance of regular follow-up with PT/INR testing, risks of discontinuation, clinical benefits of continuous and uninterrupted use of VKAs. Also, the ease of contacting the care provider through dedicated service like a 24/7 contactable phone number could have helped the patients. Prior consultation on a one-to-one basis with the care provider may also have increased the confidence as it is reflected in the data on the satisfactory questionnaire. In our study, the majority of the patients (74%) were satisfied with overall virtual care. Eighty-two percent of the patients were extremely satisfied in continuing virtual care even in the post-COVID scenario.

In our study, 57% of the patients who availed virtual care were from rural areas. WhatsApp was the most common chat platform used. A recent study by the Internet & Mobile Association of India (IAMAI) and research by Neilsen, reported that there are 227 million active internet users in rural areas in India as of November 2019 (21). This digital penetration can transform the delivery of virtual care to patients with chronic diseases in remote locations.

Preferably, patients who require VKAs, must visit in person initially and ideally should achieve at least two consecutive INRs in the therapeutic range before they could be transitioned to virtual anticoagulation clinic care for optimal patient-centered outcomes.

This pilot study has paved a path of utilizing telehealth to manage patients on chronic VKA therapy during the COVID pandemic. Though short-term results are promising, more extensive and larger multi-centric studies with a longer duration of follow-up are required to assess the feasibility and efficacy of the virtual anticoagulation clinic.

## Strengths and Limitations

The virtual anticoagulation clinic, a telehealth model that was developed during the onset of the COVID-19 pandemic to facilitate uninterrupted anticoagulation care, which could help maintain the quality of anticoagulation and minimize the risk of exposure to COVID-19. Our study has limitations such as single-center, lack of randomization, small patient population, and shorter duration of follow-up.

## Conclusions

This preliminary study showed that a virtual anticoagulation clinic can serve as a feasible alternate care model to provide uninterrupted anticoagulation care for patients on chronic Vitamin K antagonist therapy during the COVID-19 pandemic.

## Data Availability Statement

The original contributions presented in the study are included in the article/supplementary material, further inquiries can be directed to the corresponding author/s.

## Ethics Statement

The studies involving human participants were reviewed and approved by JSS Medical College and Hospital, JSS AHER. Written informed consent for participation was not required for this study in accordance with the national legislation and the institutional requirements.

## Author Contributions

All authors listed have made a substantial, direct and intellectual contribution to the work. SKS, SPSB, and OJG designed and formulated the hypothesis. RV and OJG performed data collection. SKS and OJG prepared manuscript. ND and RM reviewed the manuscript. MB, OJG, and SKS performed statistical planning and analysis. All the authors approved the manuscript for publication.

## Conflict of Interest

The authors declare that the research was conducted in the absence of any commercial or financial relationships that could be construed as a potential conflict of interest.
